# Survival outcomes among colorectal cancer patients at Kenyatta National Hospital: A retrospective cohort study

**DOI:** 10.1002/cnr2.1743

**Published:** 2022-10-25

**Authors:** Amsalu Degu, Peter N. Karimi, Sylvia A. Opanga, David G. Nyamu

**Affiliations:** ^1^ Department of Pharmaceutics and Pharmacy Practice, School of Pharmacy and Health Sciences United States International University–Africa Nairobi Kenya; ^2^ Department of Pharmacy, Faculty of Health Sciences University of Nairobi Nairobi Kenya

**Keywords:** colorectal cancer, Kenyatta national hospital, mortality, survival outcomes

## Abstract

**Background:**

Colorectal cancer is a growing burden in Africa. However, survival for patients with colorectal cancer remains low in sub‐Saharan African countries, with the poorest survival, particularly at a late stage at diagnosis. Despite this, there is a paucity of sufficient data about the survival outcomes of colorectal cancer patients in Kenya.

**Aims:**

This study aimed to determine the survival outcomes among colorectal cancer patients at Kenyatta National Hospital.

**Methods and Results:**

A retrospective cohort study was employed among 232 eligible medical records of colorectal cancer patients. Simple random sampling was used to select the medical records of the patients. The included medical records of the study participants were followed up retrospectively from the date of primary cancer diagnosis until the last visit to the hospital. All relevant data, such as sociodemographics, clinical characteristics, and outcome‐measuring parameters, were recorded in the predesigned data abstraction tool by reviewing the documented clinical records of the patients. The data were entered and analyzed using International Business Machines (IBM) Statistical Package for the Social Sciences (SPSS) version 26 software. Mean, median, standard deviation, frequency tables, and figures were used to present the data. Kaplan Meier analyses were employed to determine survival outcomes. The mean age of the study participants was 54.1 ± 13.3 years, and the majority were males (126, 54.3%). Almost a third (34.1%) of patients had evidence of disease progression despite treatment in the follow‐up period, with 7.8% showing no response to therapy and 23.6% experiencing new distant metastasis. The survival rate dwindled from the first year (87.9%) to the fifth year (45.4%), and the mortality rate was 22.8%

**Conclusion:**

There was a high mortality rate, disease progression, and distant metastasis in the last follow‐up period suggesting the need to strengthen the healthcare system by ensuring access to prevention, early diagnosis, and optimal treatment of colorectal cancer.

## 1 BACKGROUND

In 2020, an estimated 19.3 million new cancer cases and nearly 10.0 million cancer deaths occurred globally.^1^ Female breast, lung, colorectal, prostate, and gastrointestinal cancers were the leading solid malignancy across the globe.^1^ Among gastrointestinal cancers, colorectal cancer is the third most commonly diagnosed malignancy and the second leading cause of cancer death.^1^, ^2^, ^3^ Globally, 1.9 million new cases and 0.94 million deaths were reported due to colorectal cancer. The global incidence of new cases of colorectal cancer is projected to reach 3.2 million in 2040.^4^ This substantial increase in colorectal cancer incidence rates in many regions will significantly affect global health care.^5^ In many low‐income and middle‐income countries, the incidence and mortality rates are still rising rapidly.^6^

African studies have reported a growing colorectal cancer burden due to the increased prevalence of modifiable risk factors such as smoking, alcohol consumption, an unhealthy diet, and a sedentary lifestyle.^7^ Even though colorectal cancer is commonly diagnosed in an older population, there is an emerging trend of diagnosis in the younger population (aged less than 50 years).^3^ Besides, young adults with cancer face a significant burden of psychosocial and fertility issues as well as an increased risk of premature morbidity and mortality.^5^

A systematic review reported higher five‐year survival rates among colorectal cancer patients in the Iranian population.^8^ In contrast, there was a lower 5‐year relative survival in developing countries because most patients with colorectal cancer are diagnosed at an advanced stage.^9^ Survival for patients with colorectal cancer remains low in sub‐Saharan African countries, with the poorest survival occurring with individuals presenting with a late diagnosis.^10^ For instance, a previous study on the Ghananian population reported a low (16%) 5‐year survival rate among patients with colorectal cancer.^11^ There is, however, a paucity of literature about the survival outcomes of colorectal cancer patients in resource‐constrained settings such as Kenya. Therefore, this study aimed to determine the survival outcomes among colorectal cancer patients at Kenyatta National Hospital.

## 2 METHODS

### 2.1 Study design, setting, and period

A retrospective cohort study was employed between September 2021 and January 2022 at the Oncology Department of Kenyatta National Hospital. The hospital is the largest teaching and referral facility in Kenya.

### 2.2 Target population

This study targeted all medical records of adult patients with a confirmed diagnosis of colorectal cancer for 5 years, from 2016 to 2020.

### 2.3 Eligibility criteria

All medical records of adult patients (≥18 years) with a confirmed diagnosis of colorectal cancer from 2016 to 2020 with a complete medical record of diagnosis, stage of cancer, and treatment regimen were included in the study. The study excluded patients with an unconfirmed diagnosis of colorectal cancer and unclear treatment regimens.

### 2.4 Sample size determination

Yamane's formula was employed to determine the sample size^12^ and adjusted upwards by 10% to cater for incomplete medical records, giving the total sample size of 232 colorectal cancer patients.

$$n=\frac{N}{1+N*\left(e^{2}\right)}$$

Where *n* = the estimated sample size, *N* = population (445 colorectal cancer patients), *e* = the level of significance at 95% confidence level (0.05).

### 2.5 Sampling techniques

A simple random sampling by lottery method was involved in recruiting eligible medical records of colorectal cancer patients treated in the facility from January 1, 2016 to December 31, 2020.

### 2.6 Research instruments and data collection techniques

A predesigned data abstraction tool was employed in the data collection. The tool comprised sociodemographics, clinical characteristics, and outcome‐measuring parameters of colorectal cancer patients. During each day of the data collection, the list of all medical records of colorectal cancer patients was retrieved from the Health Records and Information Department of the Hospital. After assessing their eligibility using the predesigned criteria, the list of eligible patient file numbers was written on paper and reshuffled. Then, the study participants were selected randomly using the lottery method. The included medical records of the study participants were reviewed retrospectively from the date of primary cancer diagnosis until the last visit to the hospital. All relevant data, such as sociodemographics, clinical characteristics, and outcome measuring parameters, were recorded in the data abstraction tool by reviewing the documented clinical records of the patients. The American Joint Committee on Cancer staging manual was employed in staging colorectal cancer in our setting. The tumor was staged as Stages I, II, III, and IV.^13^

The proportion of patients surviving in a particular year was computed as the total number of patients surviving in each period divided by the number of patients at risk during that period. The response to treatment was determined using the modified revised guideline's Response Evaluation Criteria in Solid Tumors (RECIST).^14^ Accordingly, the response to treatment was reported as complete response (complete disappearance of all target lesions), partial response (≥30% reduction in the size of the tumor from the baseline scan), stable disease (no change in the size of the tumor) and progression of the disease (≥20% increase in tumor size or appearance of one or more new lesions) based on the interval computerized tomography (CT) scan results after treatment.

### 2.7 Data analysis

The data were analyzed using IBM SPSS version 26 software. Mean, median, and standard deviation were used to report continuous variables. Discrete variables were reported using frequency tables and figures. Kaplan Meier analyses were employed to determine survival outcomes among colorectal cancer patients.

## 3 RESULTS

### 3.1 Sociodemographic characteristics of the study participants

The mean age of the study participants was 54.1 ± 13.3 years (range: 18–95 years), with the majority (139, 59.9%) of the patients being under 60 years. Most patients were males (126, 54.3%) and self‐employed (88, 37.9%). The mean follow‐up time was 27 months, and the maximum follow‐up time was 60 months (Table 1).

**TABLE 1:** Sociodemographic characteristics of colorectal cancer patients

Variable	Frequency (%)
Age (in years)	
<60 years	139 (59.9)
≥60 years	93 (40.1)
Gender	
Male	126 (54.3)
Female	106 (45.7)
Marital status	
Single	34 (14.7)
Married	175 (75.4)
Divorced	17 (7.3)
Widowed	6 (2.6)
Educational status	
Primary	100 (43.1)
Secondary	101 (43.5)
Tertiary	30 (13)
Illiterate	1 (0.4)
Occupational status	
Self‐employed	88 (37.9)
Unemployed/Retired	43 (18.5)
Government employee	23 (9.9)
Housewife	15 (6.5)
Other^a^	63 (27.2)
Family history of cancer	
No	231 (99.6)
Yes	1 (0.4)
Mean follow‐up time (27 months)	
Maximum follow‐up time (60 months)	

^a^ Other: Driver, artisan, contractor, student, house help.

### 3.2 Clinical characteristics of the study participants

Adenocarcinoma is the most prevalent (231, 99.6%) histological type of colorectal cancer. At the time of diagnosis, most patients (58.6%) had locally advanced disease (Stages III), and 25% of patients had an advanced stage of disease (Stage IV). One‐fourth of the patients (58, 25%) had evidence of distant metastasis in different organs. The liver (26, 11.2%) and lung (17, 7.3%) were the most common sites of metastases. Multiorgan involvement was observed in 6.5%^7^ of patients. A large percentage of patients (67.2%) had no coexisting conditions. The most common concurrent co‐morbidities among colorectal cancer patients were hypertension (12, 20.3%), intestinal obstruction (18, 7.8%), and anemia (16, 6.9%) (Table 2).

**TABLE 2:** Clinical characteristics of colorectal cancer patients

Variable	Frequency (%)
Histological type of cancer	
Adenocarcinoma	231 (99.6)
Squamous cell carcinoma	1 (0.4)
Stage of cancer	
Stage I	7 (3)
Stage II	31 (13.4)
Stage III	136 (58.6)
Stage IV	58 (25)
Co‐morbidity	
Present	76 (32.8)
Absent	156 (67.2)
Number of co‐morbidities	
One	47 (20.3)
Two	22 (9.5)
≥Three	7 (3)
Type of co‐morbidity	
Hypertension	28 (12.1)
Intestinal obstruction	18 (7.8)
Anemia	16 (6.9)
Retroviral disease	8 (3.4)
Acute kidney injury	7 (3)
Hydronephrosis	4 (1.7)
Benign prostatic hyperplasia	3 (1.3)
Upper gastrointestinal bleeding	3 (1.3)
Chronic kidney disease	3 (1.3)
Stroke	3 (1.3)
Diabetes mellitus	3 (1.3)
Ascites	2 (0.9)
Congestive heart failure	2 (0.9)
Obstructive jaundice	2 (0.9)
Chronic liver disease	2 (0.9)
Peptic ulcer disease	2 (0.9)
Pneumonia	2 (0.9)
Asthma	2 (0.9)
Hemophilia	1 (0.4)
Renal calculi	1 (0.4)
Deep vein thrombosis	1 (04)
Pulmonary embolism	1 (0.4)
Arthritis	1 (0.4)
Acute liver failure	1 (0.4)
Septic shock	1 (0.4)
Distance metastasis at diagnosis	58 (25)
Liver	26 (11.2)
Lung	17 (7.3)
Liver and lung	6 (2.6)
Bone	3 (1.3)
Liver, bone, and lung	3 (1.3)
Liver and brain	2 (0.9)
Liver and bone	1 (0.4)

### 3.3 Treatment regimens of the study participants

Chemotherapy (198, 85.5%) and surgery (152, 65.5%) were the mainstays of treatment among colorectal cancer patients in our setting. Nonetheless, radiotherapy (54, 23.3%) was the least commonly used treatment approach in colorectal cancer patients (Table 3).

**TABLE 3:** Treatment regimens of colorectal cancer patients

Treatment regimen	Frequency (%)
Chemotherapy	198 (85.3)
Surgery	152 (65.5)
Radiotherapy	54 (23.3)
Symptomatic management	12 (5.2)
Chemotherapy regimens	
Folinic acid, fluorouracil, and oxaliplatin	186 (80.2)
Oxaliplatin and Capecitabine	7 (3)
Cisplatin+5‐fluorouracil	2 (0.86)
Capecitabine	2 (0.86)
Folinic acid, 5‐fluorouracil and irinotecan	1 (0.4)

### 3.4 Survival outcomes of the study participants

During the follow‐up period, distant metastasis was found in 23.6% (41) patients, of which lung (15, 8.6%) and liver (9, 5.2%) were the most prevalent sites of spread. The least common areas of spread were the bone (4, 2.3%), ovary (2, 0.9%), brain (2, 1.1%), bladder (2, 1.1%), small intestine (1, 0.6%), and pancreas (1, 0.6%).

**FIGURE 1:**
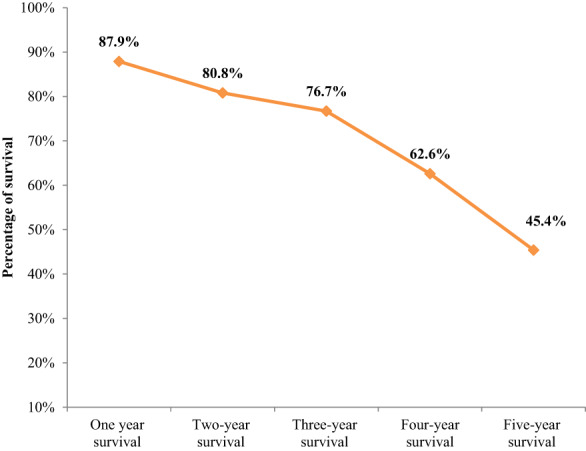
Percentage of survival rate among colorectal cancer patients

The mortality rate was 22.8% (53) among colorectal cancer patients, though 9.9% of patients had unknown outcomes. Slightly over a third (34.1%, 79) of patients had evidence of disease progression despite treatment, while 15.9% (37) and 8.6%^15^ had complete and partial responses, respectively. In addition, 7.8%^16^ of patients had no response to the treatment, while only 0.9%^2^ had stable disease (Table 4). The survival rate dwindled from the first year (87.9%) to the fifth year (45.4%) among colorectal cancer patients (Figure 1). The mean cancer‐specific survival was 26.9 ± 6.1 months, while the mean survival time from the date of cancer diagnosis to the first radiological metastasis was 20.5 ± 1.6 months. The mean survival time from the date of first radiologic metastasis until the occurrence of death or last follow‐up was 9.7 ± 1.2 months.

**TABLE 4:** Status of the response of the patients during the last follow‐up period

Status of response	Frequency (%)
Progression of the disease	79 (34.1)
Complete response	37 (15.9)
Partial response	20 (8.6)
Non‐response	18 (7.8)
Death	53 (22.8)
Unknown	23 (9.9)
Stable disease	2 (0.9)

The study showed that patients aged ≥60 years had a shorter median survival time than those aged <60 years (33.0 ± 9.6 months vs. 48.0 ± 17.3 months). Patients with colorectal cancer and co‐morbid conditions exhibited a reduced median survival time (34.0 ± 6.9 months) compared with those without co‐morbid disorders (48.0 ± 15.5 months). The median survival time of patients with distant metastasis was smaller than patients without metastasis.

**FIGURE 2:**
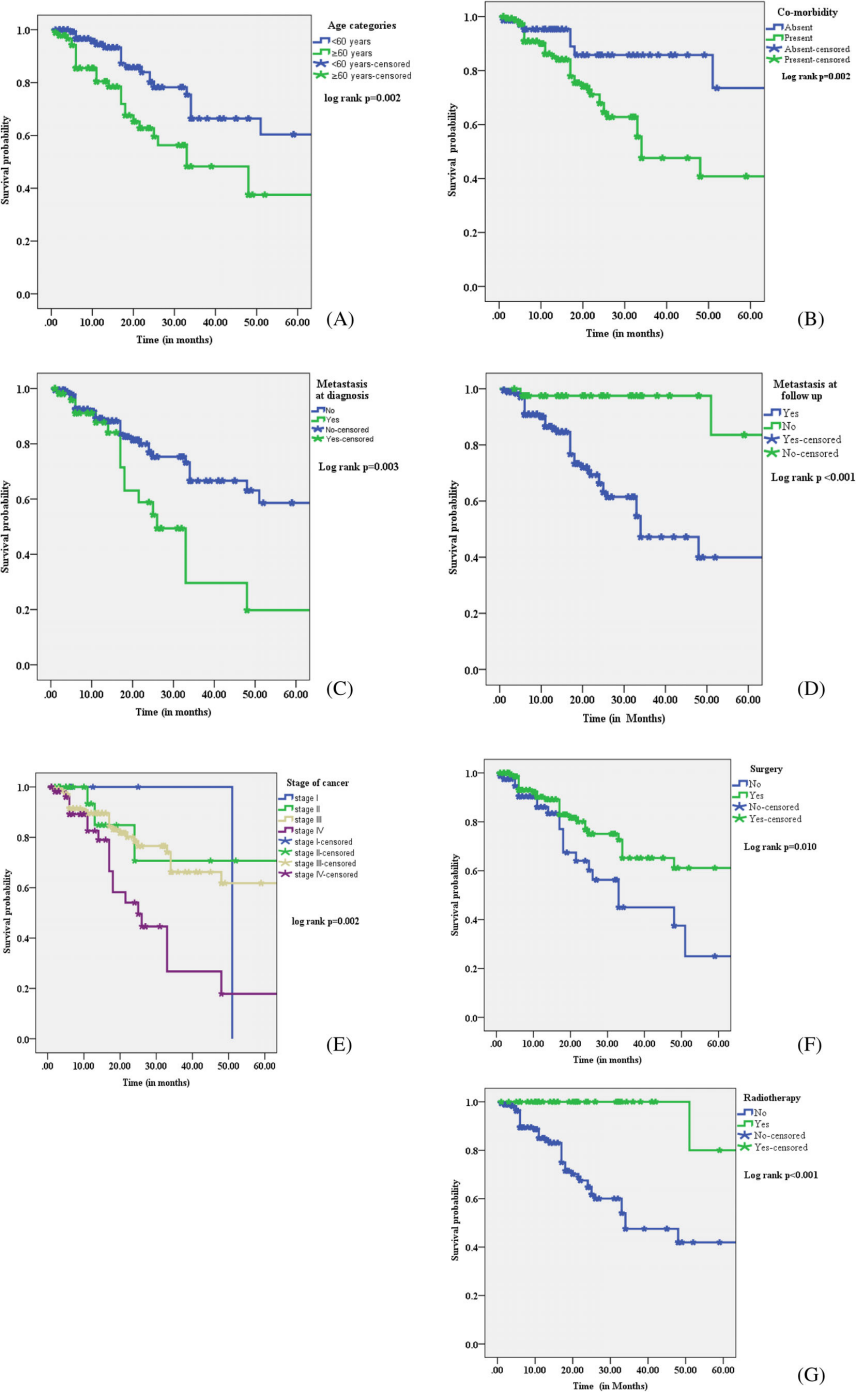
Kaplan–Meier survival curve of colorectal cancer patients based on age categories, co‐morbidity, metastasis, tumor stage, and treatment regimen

Patients with Stage IV tumors (25.8 ± 5.7 months) had a shorter median survival time than those with Stage I (51.0 ± 2.3 months), II (48.0 ± 4.7 months), and III (32.8 ± 5.7 months) of the disease. Patients who underwent surgical intervention and radiotherapy had a longer median survival time than those without surgical intervention and radiotherapy, respectively. However, gender and chemotherapy did not show significant differences in median survival time in colorectal cancer patients in the study setting (Table 5, Figure 2).

**TABLE 5:** Median survival time among colorectal cancer patients

Variables	Median survival time (months) ± standard error (95% CI)	Log‐rank test (*p*‐value)
Age (years)		.002*
<60 years	48.0 ± 17.3 (43.9–112.0)	
≥60 years	33.0 ± 9.6 (14.1–51.9)	
Gender		.492
Male	48.0 ± 10.9 (56.4–99.5)	
Female	48.1 ± 15.5 (47.6–108.4)	
Co‐morbidity		.002*
Present	34.0 ± 6.9 (20.4–47.6)	
Absent	48.0 ± 15.5 (47.6–108.4)	
Stage of cancer		.002*
Stage I	51.0 ± 2.3 (23.2–62.5)	
Stage II	48.0 ± 4.7 (70.7–131.6)	
Stage III	32.8 ± 5.7 (21.7–43.9)	
Stage IV	25.8 ± 5.7 (21.7–43.9)	
Distant metastasis at diagnosis		.003*
Yes	48.0 ± 19.4 (39.9–116.0)	
No	26.0 ± 4.0 (18.2–33.8)	
Distant metastasis in the follow‐up period		<.001*
Yes	34.0 ± 6.7 (20.9–47.1)	
No	48.0 ± 15.5 (47.6–108.3)	
Treatment regimen		
Chemotherapy		.778
No	51.0 ± 16.1 (47.6–108.4)	
Yes	48.0 ± 15.4 (46.4–109.6)	
Surgery		.010*
No	33.0 ± 11.7 (10.1–55.9)	
Yes	48.0 ± 15.5 (47.6–108.4)	
Radiotherapy		<.001*
No	34.0 ± 7.5 (19.3–48.7)	
Yes	48.0 ± 15.6 (47.6–108.5)	

* Statistically significant *p*‐value ≤.05, CI: Confidence interval.

## 4 DISCUSSION

The burden on colorectal cancer is high in African countries.^7^ The present study has highlighted the extent of survival outcomes among the patients in the largest referral health facility in East and Central Africa.

The study has established that the mean cancer‐specific survival was 26.9 ± 6.1 months which was higher than the Ghanian study (15 months),^11^ Ethiopian (34.8 and 21 months),^17^, ^18^ and Cameroonian studies (43 months).^16^ These disparities may be due to differences in early screening programs, stage of disease at diagnosis, and referral level of healthcare facilities.

The present study has revealed a lower survival rate (mortality at 22.8%), tallying with the literature that documented that the survival of colorectal cancer patients is poor in sub‐Saharan African countries.^10^ Ethiopian studies also reported a higher incidence of mortality (34.8%) in colorectal cancer patients.^18^

Despite the advancement of colorectal cancer management, cancer‐specific survival remains low in sub‐Saharan African countries.^10^ This study revealed that colorectal cancer patients' survival rates dwindled from the first year (87.9%) to the fifth year (45.4%). Contrastingly, a study in Ghana (16%) and Indonesia (8.7%) demonstrated very low cancer‐specific survival among colorectal cancer patients.^11^, ^19^ This disparity could probably be linked to the difference in the advancement of screening, diagnostic and treatment facilities.^15^ The low probability of survival rate could be linked to the late diagnosis and delayed treatment in the study setting due to inadequate cancer treatment centers in resource‐limited settings. Low and middle‐income countries experience a higher burden and low 5 years survival rates among colorectal cancer patients compared with developed countries.^10^ Previous studies demonstrated that late diagnosis had the worst survival in colorectal cancer patients.^20^, ^21^ Inadequate health coverage may restrict access to optimal diagnostic and therapeutic care, which can negatively affect the desired treatment outcomes.

Most patients were aged <60 years and had locally advanced (58.6%) and advanced stage (25%) at the time of diagnosis. This finding agrees with previous studies in low and middle‐income countries, which reported a relatively higher percentage of patients aged <60 years and in the advanced stage at diagnosis.^22^ Moreover, an African study revealed that the late stage and younger age (<50 years) at presentation were common among colorectal cancer patients.^10^ The present study showed that 23.6% of patients had experienced new distant metastasis in the follow‐up period. A previous related study reported the incidence of distant metastasis was 17.3% among colorectal cancer patients.^23^ Another study reported that nearly half of the patients developed distant metastasis after resecting the primary tumor with an abysmal prognosis.^24^ Lung and liver were the most prevalent sites of metastasis, collaborating with similar studies.^19^, ^25^, ^26^ These patients have a much lower chance of achieving long‐term remission than those with localized cancer since metastatic tumors are mostly unresponsive to the existing therapies.

In the last follow‐up period, 34.1% of patients had evidence of disease progression despite treatment. In addition, 7.8% of patients had no response to the treatment suggesting that patient‐specific interventional strategies should be implemented to circumvent the high rate of disease progression and nonresponse to therapy among colorectal cancer patients.

### 4.1 Strengths and limitations of the study

This was the first study to evaluate survival outcomes for colorectal cancer patients in the study setting. However, due to the retrospective nature of the study, the validity of the data will be affected by the accuracy of the documentation in the study setting. Moreover, because it was conducted in a single healthcare facility, it may not be replicated in the entire population.

## 5 CONCLUSIONS

There was a high mortality rate (22.8%), disease progression (34.1%), and nonresponse (7.8%) to the treatment among colorectal cancer patients in our setting. Therefore, awareness creation of educational programs about early screening, preventable risk factors, and equitable access to optimal diagnostic and treatment care is paramount to improving the survival of colorectal cancer patients in Kenya.

## AUTHOR CONTRIBUTIONS

**Amsalu Degu:** Conceptualization (equal); data curation (equal); formal analysis (equal); funding acquisition (equal); investigation (equal); methodology (equal); project administration (equal); resources (equal); software (equal); supervision (equal); validation (equal); visualization (equal); writing – original draft (equal); writing – review and editing (equal). **Peter N Karimi:** Conceptualization (equal); data curation (equal); formal analysis (equal); funding acquisition (equal); investigation (equal); methodology (equal); project administration (equal); resources (equal); software (equal); supervision (equal); validation (equal); visualization (equal); writing – original draft (equal); writing – review and editing (equal). **Sylvia A Opanga:** Conceptualization (equal); data curation (equal); formal analysis (equal); funding acquisition (equal); investigation (equal); methodology (equal); project administration (equal); resources (equal); software (equal); supervision (equal); validation (equal); visualization (equal); writing – original draft (equal); writing – review and editing (equal). **David G Nyamu:** Conceptualization (equal); data curation (equal); formal analysis (equal); funding acquisition (equal); investigation (equal); methodology (equal); project administration (equal); resources (equal); software (equal); supervision (equal); validation (equal); visualization (equal); writing – original draft (equal); writing – review and editing (equal).

## CONFLICT OF INTEREST

The authors have stated explicitly that there are no conflicts of interest in connection with this article.

## ETHICS STATEMENT

The study protocol was approved by the Kenyatta National Hospital/University of Nairobi Ethics and Research Committee (Approval No: P195/03/2021). Before data collection, official permission was obtained from the Health Records and Information Department of the hospital. The names and addresses of the patients were not recorded during the data collection to ensure the confidentiality of patient data.

## PATIENT CONSENT STATEMENT

The ethics committee waived the participants' informed consent requirements due to the retrospective nature of the study design.

## Data Availability

The data sets used and/or analyzed during the current study will be obtained from the corresponding author of this project.
